# Accumulation of material and lifestyle problems among daily smokers in Norway 1999 to 2013 - a cross-sectional analysis

**DOI:** 10.1186/s12889-016-3465-3

**Published:** 2016-08-12

**Authors:** Gunnar Sæbø, Elisabeth Kvaavik

**Affiliations:** Norwegian Institute of Public Health (FHI), P.O. Box 4404, Nydalen, N-0403 Oslo Norway

**Keywords:** Smoking, Lifestyle, Accumulation of problems, Marginalisation

## Abstract

**Background:**

International studies have found that smoking is increasingly concentrated among lesser-privileged individuals and marginalised groups, indicating a possible rise in daily smokers’ accumulated problem burden. The study asks whether material shortages and occurrence of behaviours related to poor health are increasing among daily smokers in Norway, and whether the time trends differ between daily smokers on the one hand, and occasional and non-smokers on the other.

**Method:**

The study used data acquired by biennial cross-sectional surveys from 1999 to 2013 of the adult (i.e. over 15) Norwegian population. Time trends in individual and accumulated material and lifestyle problems among daily smokers and non-daily and non-smokers combined were assessed using logistic regression analyses for men and women separately.

**Results:**

The accumulation of problems in any isolated survey is higher among daily smokers than other respondents. Over the longer term, however, there are few signs of decline in any group, except in regards to frequent alcohol drinking, which increased in all studied groups. The only problem factor differentiating daily smokers from occasional smokers/non-smokers that did change during the period was quality of diet. While problem accumulation declined in all but one group, i.e., male daily smokers, the difference between them and the group of occasional smokers and non-smokers was not significant.

**Conclusion:**

Daily smokers are generally worse off than occasional smokers and non-smokers combined. However, the accumulation of material problems and health-risk behaviours by daily smokers and occasional smokers/non-smokers did not change significantly and all groups had fewer problems in 2013 than in 1999.

## Background

Smoking is the single most preventable cause of disease. While smoking prevalence has declined in many Western countries in recent decades, it is important to strengthen tobacco control efforts – to prevent adolescents from taking up smoking, prevent the tobacco industry from promoting its products and to facilitate smoking cessation. Although large segments of the population have already given up smoking, and a small number of smokers succeed in quitting every year, success in quitting differs markedly among social groups. For instance, better educated smokers are more likely to succeed [1–3]. In recent decades, the decline in smoking rates among more educated groups has been faster than among less educated groups, both in Europe and the US, although inter-country variation is notable [2, 4, 5]. This decline is widening the gap in smoking rates among people with different educational achievements. In Norway, as in many other developed countries, an important objective of tobacco control policy has been to narrow the social inequality gap in smoking; success thus far has only been moderate [3, 4, 6–8]. Social inequalities in smoking-related diseases will likely persist and might even increase in the future [4–9].

### The marginalisation of smokers

It has been suggested that various socioeconomic position (SEP) groups have different levels and sources of resistance to tobacco control [10–12], resulting in varying uptake and quitting rates, eventually leading to different smoking rates among persons in different social positions. As the findings of several studies suggest, smoking and other material and lifestyle problems (such as diet and physical activity) are more likely to accumulate among marginalised groups, i.e., low SEP groups [13–15]. The accumulation of problems among daily smokers may therefore become more prominent over time [12, 16]. A recent study from England found that smoking prevalence decreased across the board between 2001 and 2008 (as a result of increasing numbers of never smokers rather than of former smokers because of declining rates of smoking uptake), apart from among the multiple disadvantaged (defined as meeting at least four of the following seven criteria: routine or manual occupation; deprived neighbourhood; single parent; no car; rented accommodation; low income; and unemployment). Disadvantage declined among non-smokers, but not among smokers [17].

As daily smoking is more and more associated with passive and unhealthy lifestyles, smoking is increasingly considered a symbol and marker of social exclusion. This has triggered a debate on the marginalisation of smokers [18, 19]. Empirically, smoker marginalisation can be approached in two different (not mutually exclusive) ways. First, it may refer to smokers being overrepresented (and increasingly so over time) in groups that are already marginalised, such as aboriginal groups, people with mental illness or substance abuse problems [20]. Second, it can refer to increasing marginalisation of smokers as a deviant group in relation to the average population [21]. While smoking in the 1970s was perceived as a normal activity, and smokers shared many of the social and cultural characteristics of non-smokers, current smokers (daily smokers in particular) have been “denormalised” and are more likely to be poor and less well educated in comparison to non-smokers. Occasional, or non-daily, “social” smokers are found to differ from daily smokers with regard to demographics, socioeconomic status and material and lifestyle problems in that they are often younger and more privileged in terms of educational and material resources [22–24]. Occasional smokers also often deny an identity as a smoker [25, 26]. As a group, occasional smokers tend to have more in common with non-smokers than daily smokers [22, 27].

Many of the remaining established daily smokers in the population are unable (due to nicotine addiction) – or do not want – to quit smoking. The concept of “hard-core smokers” is sometimes applied to designate this group [28]. Even if some of the research on these smokers does touch on processes of social marginalisation, it is essentially concerned with the “hardening” of the smoker group regarding nicotine addiction, smoking behaviour and motivation to quit. While socio-demographic background factors are often accounted for, studies of hard-core smokers do not consider the social and lifestyle context of smoking as their primary object of study.

In fact, there are few (if any) empirical studies addressing whether inequality between smokers and non-smokers with regard to occurrence of material *and* lifestyle problems has increased. Nor do we know much about the accumulation of problems among daily smokers over time. Different rates of problem accumulation may increase social inequalities as well as differences in lifestyles between daily smokers and occasional smokers/non-smokers, leaving daily smokers an increasingly marginalised group, possibly on the fringes of society.

In this article, we investigate whether we can detect signs of growing marginalisation, in terms of increasing accumulation of material and lifestyle problems at the individual level, in the group of remaining daily smokers in the Norwegian population. Admittedly, this approach does not address marginalisation directly; that is, as actions made at the interpersonal and structural levels reducing the chances of achieving wealthier or healthier lives. Drawing on the social indicators approach, we rather explore respondents’ level of living in its totality [29] by combining indicators of an “objective” material situation with more “subjective” lifestyle components relevant to public health. By “material problems”, we mean financial and housing insufficiencies relative to societal norms and/or statistical averages in the population. By “lifestyle problems”, we refer to “chosen” behaviours that are generally agreed to be troublesome to health (unhealthy dietary habits, high alcohol consumption, minimal physical exercise). In addition, we address occurrence of problems with regards to self-rated health, social network and education. Our main concern is whether the overall level of living (including both material and cultural life styles dimensions) have worsened for smokers over time.

### Research hypothesis

The current study examines changes in material and lifestyle problems, health risk behaviours and poor self-rated health among daily smokers in Norway between 1999 and 2013 compared to occasional and non-smokers (which for the purposes of the study were collapsed into a single group). We hypothesise that daily smokers are worse off than non-daily smokers or non-smokers, and that problems increase and accumulate more markedly among daily smokers in Norway over time.

## Methods

### Data

We performed secondary analyses of data stemming from *Norwegian Monitor*, a biennial population based survey of Norwegian lifestyles and opinions, organised by the public opinion agency IPSOS. Data was collected from 1999 to 2013.

Respondents aged 15 years and older were recruited by way of probability sampling; telephone records were used to draw a random sample of the Norwegian population. The exact size of the gross sample is not known due to uncertainty about the status of the telephone numbers (live, dead). Those with operational telephone numbers and who agreed to, and participated in, a short telephone interview, were also invited to take part in the main part of the survey, thus constituting the eligible sample for the current study. Those who responded to the questionnaire constitutes the analytical sample in the analyses presented here. Out of those constituting the eligible sample, 52 % returned questionnaires in 2002; the return rate fell steadily thereafter to 37 % in 2013 [30]. Increased attrition in recent years has been met by expanding the gross samples. The questionnaire was self-administered and contained around 3000 questions, including standardised measures such as SRH-5 (self-rated health) and one item from AUDIT.

Identical sampling procedures were used for all years included in the current study (1999, 2001, 2003, 2005, 2007, 2009, 2011 and 2013) and the analytical sample size varied between 3429 and 4124 subjects (50–51 % female) in the time period. The number of daily smokers varied between 1116 in 1999 and 490 in 2013. To strengthen representativeness, the sample was weighted for gender, age and region of the country (Oslo and the Eastern, Western, Central and Northern parts of Norway).

### Ethical clearance process

No sensitive information was collected; hence, there were no need for any committee approval. Respondents consented to participation twice: first, when they agreed to participate in the introductory telephone interview; second, when they completed and returned the postal questionnaire. The data set was anonymised before being submitted to the authors for analysis. It is not possible to identify respondents by way of combining variables.

### Measures

Smoking status was assessed by asking the question “Would you say that you smoke daily, non-daily/occasionally or never?” This is a standard measure of smoking behaviour, which is widely applied in the tobacco control community. In the analyses, daily smokers were compared to occasional smokers and non-smokers (the two latter categories were collapsed).

To assess material and lifestyle problems, we applied ten indicators, each recoded as a dichotomous “problematic/non problematic” status. All these measures are customary in public health research.

- Occupational position was assessed by asking about the person’s employment status, where the response alternatives “unemployed”, “on social benefit” and “married and unemployed” (the latter suggesting economic dependence on the spouse) were combined to represent problematic status. This measure was designated *not in work* [31].
- Type of housing and house ownership was assessed by combining information about accommodation type (house, flat, bedsit) and whether the respondent rented or owned the property. In Norway, in contrast to many other countries, it is common for people to be homeowners [32]. *Renting a flat or bedsit* was therefore defined as the problematic outcome [33].
- Personal economic situation was assessed by asking “What is the current financial situation of your household?” with the response categories “income is not adequate” and “we/I need to use our/my savings to manage” combined to constitute the outcome *perceived poor economy*. This measure is often applied in research on poverty and well-being [34].
- Respondents with *elementary school only* (maximum 9 years) were defined as having brief/minimal education – i.e. a problem status [33].
- A combination of non-membership of seven named associations/organisations and meeting friends less than weekly were combined to represent *small social network* [35].
- *Low intake of fruit and vegetables* was defined as having fruit and vegetables twice a week or less [36]. This cut off is lower than the recommended intake of 5 servings a day because only half the population eat fruit and vegetables daily while very few eat 5 servings a day [37].
- *High intake of fast food* was defined as consumption of hot dogs (bought at fast food outlets or petrol stations) and/or eating chips at least once a week [38, 39].
- *Drinking alcohol often* (defined by the respondents by answering the question “How often do you drink alcohol?” with response categories “often”, “now and then”, “seldom”, “never”) was used as the outcome for potentially problematic drinking [40]. This measure is part of the Alcohol Use Disorder Identification Test - AUDIT. (No other items from AUDIT were included in the questionnaire).
- *Never exercising* (registered by the question “How often would you say you exercise, i.e. are physically active?” with eight response categories ranging from “never” to “at least once a day”) were used as the outcome for low physical activity [41].
- Self-rated health was recorded by asking the respondents to grade their health as “very good”, “good”, “moderate”, “poor”, or “very poor”. This is an established indicator to assess subjective health (SHR-5) [42]. The last two response alternatives were defined as indicators of *poor self-rated health* [43].

While some of the “cut-offs” are self-explanatory (such as “not in work” and “elementary education only”), others were designed with a view to using a strict definition of what constitutes a problematic situation (and to keep the share with “problems” as less than 20 % on each indicator, to reduce overall distribution of problems in the sample). When selecting these indicators, we also looked at missing responses, which we attempted to keep at a minimum – i.e. as low as possible.

All problems (not in work, renting flat or bedsit, perceived poor economy, elementary school only, small social network, low fruit and vegetable intake, high fast food intake, regular alcohol consumption, never exercising, poor self-rated health) were given a score of one and also summed up in an additive index to represent accumulation of material and lifestyle problems (ranging from 0 to 10). In addition, a score of four or more was defined as a condition of accumulated substantial problems.

### Statistical analysis

All data analyses were performed separately for men and women using weighted data. Age-adjusted percentages for each variable by year and smoking group were computed using ANOVA. Trends in material and lifestyle problems were analysed for each smoking status group (daily smokers, and occasional and non-smokers combined) and adjusted for age, using logistic regression analyses with time as the independent variable. To test whether the time trends were similar in the two groups, data was analysed in multivariate models using logistic regression with age, smoking, time, and the interaction term time*smoking as covariates. Chi Square tests were used to compare daily smokers and occasional and non-smokers combined with regard to accumulated problems for each year and T-test to compare smoking groups with regards to mean problem score for all individual years.

Sensitivity analyses were performed to test whether results differed when daily smokers were contrasted with occasional smokers, non-smokers and occasional smokers/non-smokers combined, respectively. They did not, which is why we decided to present results using occasional smokers and non-smokers combined. Nor did additional sensitivity analyses that excluded the youngest respondents (25 years of age or younger) provide results that differ substantially from the main results.

Further sensitivity tests were performed to investigate whether the occurrence of missing categories influenced results significantly. The indicators we applied usually had a missing percentage of 0.2–2.0, with the highest noted exception being 4.1 %. Missing shares did not vary between daily smokers and others, and none were found to influence the results significantly. Nor did the missing share of these indicators change over time.

With regard to the additive index, correlation matrices were investigated to check whether some problems tended to be more strongly correlated with others, suggesting strong partial accumulation (see Table 1). No such patterns were found. The only exceptions worth mentioning were “not in work” and “poor health” (rho = .25 among men, rho=. 28 among women) and “not in work” and “poor personal economy” (rho = .13 among men, rho = .12 among women), none of which are particularly strong associations. Thus, we are assured that the indicators measure different empirical phenomena, whose patterns of aggregation can be analysed by way of an additive index.

**Table 1:** Nonparametric correlations between the outcome problems, men (upper right) and women (lower left). Spearman’s rho

	1.	2.	3.	4.	5.	6.	7.	8.	9.	10.
1. Not working		0.03**	0.05**	0.13**	0.10**	0.25**	0.05*	−0.01	−0.04**	0.08**
2. Renting home	−0.02*		−0.07**	0.13**	−0.06**	0.00	0.07**	0.06**	0.06**	0.00
3. Small social network	0.06**	−0.07**		0.02*	0.02*	0.06**	0.02**	−0.05**	−0.02*	0.10**
4. Poor economy	0.12**	0.11**	0.00		0.01	0.09**	0.06**	0.05**	0.01	0.04**
5. Elementary school only	0.10**	−0.04**	0.01	0.03**		0.05**	0.05**	0.03**	−0.09**	0.07**
6. Poor self-rated health	0.28**	0.01	0.05**	0.10**	0.05**		0.04**	0.00	0.00	0.11**
7. Low fruit and vegetable intake	0.03**	0.07**	0.02**	0.07**	0.04**	0.03**		0.08**	0.02**	0.11**
8. High fast food intake	−0.01	0.04**	−0.03**	0.06**	0.04**	0.02**	0.07**		0.04**	0.05**
9. Drinking alcohol often	−0.05**	0.03**	−0.01	−0.01	−0.10**	−0.03**	−0.01	0.00		−0.01
10. No exercise	0.07**	0.01	0.09**	0.05**	0.12**	0.10**	0.08**	0.04**	−0.03**	

*P < 0.05, **P < 0.01

Sensitivity checks were performed to check alternative cut offs for the dichotomised version of the index. A score of three gave similar statistical patterns as four, but at a higher rate. A score of five could was not applicable, as very few persons had that many problems.

Analyses were performed using SPSS version 23.0 [44].

## Results

**Fig. 1:**
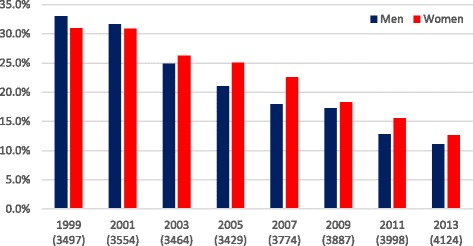
Prevalence of daily smoking from 1999 to 2013. Weighted data, total number of respondents each year in parentheses

The prevalence of daily smoking has more than halved among men and women between 1999 and 2013 from over 30 to under 15% (Fig. 1). For other descriptive statistics, see Table 2.

**Table 2:** Descriptive statistics for men and women in 1999 and in 2013. Percentages

Variable	Categories	1999		2013	
		Men (*n* = 1717^a^)	Women (*n* = 1779^a^)	Men (*n* = 2061^a^)	Women (*n* = 2063^a^)
Job position	Unskilled manual worker	7.9	7.8	6.2	5.0
	Skilled manual worker	16.7	11.7	17.3	17.0
	Chief executive	4.7	2.9	5.3	3.0
	Skilled, non-manual, leader	14.4	6.1	13.5	7.3
	Middle manager	8.9	12.5	9.9	12.9
	Manager owner	6.9	3.4	7.3	3.9
	Student/apprentice	10.8	11.2	12.2	12.1
	*Unemployed* ^b^	*1.0*	*1.1*	*1.3*	*1.4*
	Retirement pensioner	14.3	17.7	14.3	16.0
	*Benefit recipient*	*5.1*	*9.4*	*3.8*	*6.3*
	*Married and not employed*	*0.3*	*1.7*	*0.4*	*1.1*
	Other	9.2	14.5	8.4	13.9
Type of housing and ownership	Own a house, single unit	56.0	54.2	57.9	56.2
	Own a 2–4 family house or row house	14.2	14.8	15.0	13.5
	Own a flat or bedsit	11.2	12.2	12.6	15.3
	Own other type of home	1.6	2.4	1.0	1.3
	Rent a house, single unit	5.2	5.4	2.9	3.4
	Rent a 2–4 family house or row house	4.1	3.9	2.7	1.5
	*Rent a flat or bedsit*	*5.5*	*5.1*	*6.7*	*7.2*
	*Rent other type of home*	*2.2*	*2.1*	*1.1*	*1.7*
Social network	*No membership, never meeting friends*	*2.6*	*2.4*	*3.1*	*1.8*
	*No membership, meeting friends less than weekly*	*19.3*	*22.8*	*20.3*	*22.2*
	No membership, meeting friends at least weekly	23.9	31.3	22.0	25.2
	1 membership, never meeting friends	2.2	1.0	1.8	1.0
	1 membership, meeting friends less than weekly	13.7	11.6	16.6	13.4
	1 membership, meeting friends at least weekly	23.5	19.5	18.1	18.9
	2 memberships or more, never meeting friends	0.5	0.1	0.4	0.3
	2 memberships or more, meeting friends less than weekly	6.4	4.1	8.2	8.0
	2 memberships or more, meeting friends at least weekly	7.9	7.2	9.5	9.2
Personal economic situation	*My/our income is not adequate*	*4.9*	*7.2*	*3.5*	*3.1*
	*I/we need to use my/our savings*	*4.2*	*7.6*	*3.7*	*5.4*
	I/we just manage	37.5	42.4	25.4	27.0
	I/we manage and can save some money	45.0	37.9	52.6	51.9
	I/we manage and can save quite a bit	7.0	3.7	16.3	12.0
	Don’t know	1.4	1.3	0.8	0.5
Education	*Elementary school (7–9 years)*	*6.2*	*9.2*	*2.8*	*2.0*
	Lower secondary/comprehensive school (9–10 years)	16.9	16.8	9.3	9.4
	Upper secondary/high school (12–13 years)	38.9	39.3	30.7	28.5
	College/university (14+ years)	38.0	34.7	57.3	60.1
General, self-rated health	Very good	20.9	20.9	27.7	26.0
	Good	46.8	44.5	45.6	47.0
	Moderate	27.1	27.6	21.4	20.7
	*Poor*	*4.7*	*6.6*	*5.0*	*5.8*
	*Very poor*	*0.5*	*0.5*	*0.2*	*0.5*
Fruit and vegetable intake	*Seldom/never*	*5.7*	*3.1*	*4.4*	*1.9*
	*1–2 times per week*	*4.5*	*2.2*	*2.9*	*1.2*
	3–6 times per week	17.4	9.7	14.9	9.0
	At least daily	72.4	84.9	77.7	87.9
Fast food intake	Seldom/never	5.2	9.6	2.4	7.1
	Less than weekly	76.4	83.6	86.9	90.0
	*1–4 times per week*	*17.2*	*6.8*	*10.2*	*2.8*
	*5 times or more per week*	*1.2*	*0.1*	*0.4*	*0.0*
Drinking alcoholic drinks	*Often*	*21.1*	*10.8*	*25.9*	*19.0*
	Now and then	49.9	49.7	48.3	45.0
	Seldom	21.1	29.7	18.3	26.9
	Never	7.9	9.8	7.5	9.1
Exercising	*Never*	*13.4*	*11.7*	*6.6*	*5.9*
	Less than once each fortnight	14.4	13.3	9.5	9.0
	Once each fortnight	7.2	6.1	7.0	5.2
	Once a week	16.7	17.4	14.4	14.5
	Twice a week	19.6	21.9	22.0	23.7
	3–4 times/week	18.2	18.2	27.0	26.9
	5–6 times/week	6.3	7.0	8.7	9.9
	At least once daily	4.1	4.3	4.9	4.9
All 10 problems	0	25.6	29.7	34.0	37.3
	1	35.7	34.6	35.9	38.0
	2	22.6	21.6	19.9	16.6
	3	10.5	10.1	7.2	6.1
	*4*	*4.1*	*2.8*	*1.6*	*1.7*
	*5*	*1.2*	*1.0*	*0.9*	*0.3*
	*6*	*0.2*	*0.2*	*0.4*	*0.0*
	*7*	*0.1*	*0.1*	*0.0*	*0.0*
	*8* ^c^	*0.0*	*0.0*	*0.0*	*0.0*

^a^Maximum N, varies depending on the variable due to missing values

^b^Category/ies written with *italics* within each variable (were combined to) represent the problematic outcome in the analyses

^c^No women and only 1 man (in 2007) had 8 problems

**Fig. 2:**
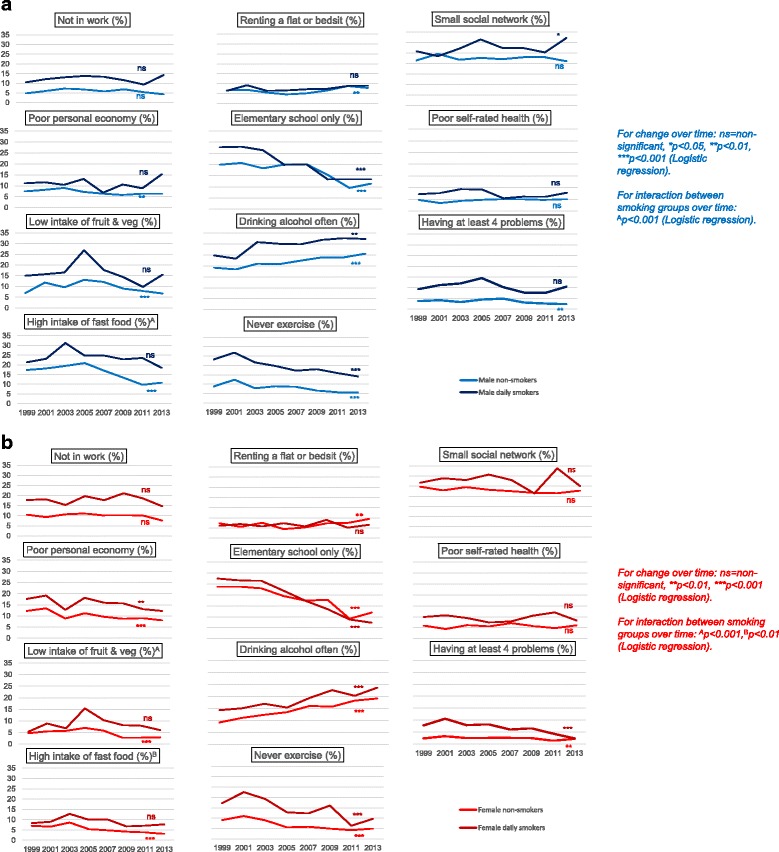
**a** Material and lifestyle problems among daily smokers and occasional and non-smokers combined between 1999 and 2013. Age-adjusted percentages (ANOVA). Men. **b** Material and lifestyle problems among daily smokers and occasional and non-smokers combined between 1999 and 2013. Age-adjusted percentages (ANOVA). Women

Prevalence of most individual problems decreased or stagnated during the period from 1999 to 2013 for both men (Fig. 2a) and women (Fig. 2b). An exception is “drinking alcohol often”, with frequency increasing significantly among male and female daily smokers and occasional and non-smokers combined between 1999 and 2013. Nearly all problems were more prevalent among daily smokers than among occasional and non-smokers combined for each year; the same also applies to regular use of alcohol (*P*-values not shown).

However, only “low intake of fruit and vegetables” for women and “high intake of fast food” for both men and women changed differently between daily smokers and occasional and non-smokers combined. While daily smokers did not change their dietary behaviour, the diets of occasional and non-smokers improved over the years (Fig. 2a/b). The likelihood of having a minimum of four problems declined significantly in all groups, with the exception of male daily smokers.

**Fig. 3:**
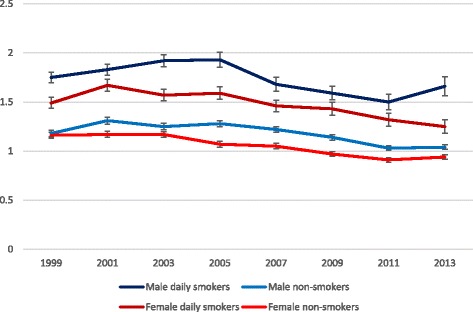
Mean problem score (with SE) 1999–2013. Mean problem score differ between smoking groups each individual year and all years combined (*P* < 0.001, T-test)

The distribution of number of problems among daily smokers and occasional/non-smokers combined for each year is shown in Table 3. While the most commonly observed pattern among the latter group was 0 and 1 problem, it was 1 and 2 problems among daily smokers. (This especially applied to men). Also, the tendency for daily smokers to more often have a score of 4 problems or more suggests a greater accumulation of problems among daily smokers. The same pattern is illustrated in Fig. 3, which shows the mean scores for each group over time.

**Table 3:** Proportions (%) of daily smokers (DS) and occasional and non-smokers combined (ONS) having between none and ten problems for all years between 1999 and 2013. Number of problems differ between smoking groups each individual year and all years combined (*P* < 0.001, Chi-square test)

No. of problems	1999		2001		2003		2005		2007		2009		2011		2013		All years	
Men (n)	ONS (1151)	DS (565)	ONS (1190)	DS (551)	ONS (1276)	DS (420)	ONS (1328)	DS (354)	ONS (1521)	DS (334)	ONS (1592)	DS (333)	ONS (1731)	DS (254)	ONS (1826)	DS (229)	ONS (11 615)	DS (3041)
0	30.1	16.4	27.7	14.5	28.8	13.0	28.1	14.6	30.4	17.3	31.4	19.8	34.8	23.7	35.2	25.1	31.2	17.1
1	37.6	31.8	33.8	31.0	34.8	27.0	35.6	30.0	34.6	36.2	37.3	33.8	38.0	30.7	37.2	25.5	36.2	30.9
2	21.2	25.4	23.8	29.2	23.3	29.5	21.5	24.5	23.1	21.2	21.3	24.1	19.2	26.7	19.2	25.7	21.4	26.1
3	7.5	16.8	10.6	14.0	9.7	18.8	10.4	16.5	7.3	14.9	7.3	14.7	5.8	12.0	6.4	14.1	7.9	15.5
4	2.9	6.3	2.6	7.4	2.7	8.8	3.5	8.4	3.6	8.4	1.7	6.3	2.0	4.9	1.4	3.2	2.5	7.0
5	0.4	2.8	1.0	2.1	0.7	2.7	0.7	4.3	0.8	1.5	0.8	0.3	0.2	1.7	0.5	3.9	0.6	2.4
6	0.2	0.1	0.3	1.4	0.0	0.2	0.1	1.6	0.1	0.0	0.2	0.6	0.0	0.3	0.2	2.4	0.2	0.8
7	0.1	0.3	0.1	0.4	0.0	0.0	0.0	0.0	0.0	0.2	0.1	0.4	0.0	0.0	0.0	0.0	0.0	0.2
8^a^	0.0	0.0	0.0	0.0	0.0	0.0	0.0	0.0	0.0	0.3	0.0	0.0	0.0	0.0	0.0	0.0	0.0	0.0
Women (n)	ONS (1228)	DS (548)	ONS (1249)	DS (559)	ONS (1301)	DS (464)	ONS (1302)	DS (438)	ONS (1479)	DS (432)	ONS (1596)	DS (358)	ONS (1697)	DS (313)	ONS (1796)	DS (261)	ONS (11 648)	DS (3374)
0	31.9	24.5	30.3	21.3	28.8	22.1	34.5	21.3	36.1	22.0	38.0	26.4	40.8	26.6	38.7	27.9	35.4	23.6
1	35.3	33.0	38.8	32.0	38.7	31.2	36.7	33.5	35.8	36.6	36.9	32.1	35.6	36.6	38.1	37.4	37.0	33.7
2	21.1	22.5	18.8	21.7	21.9	25.6	19.5	24.3	19.3	25.3	17.7	23.0	17.0	21.5	16.2	19.7	1.87	23.1
3	9.0	12.5	9.0	14.1	7.9	12.3	7.1	12.2	6.1	9.5	5.2	12.2	5.0	10.8	5.2	12.4	6.6	12.1
4	2.0	4.6	2.3	7.1	2.4	7.1	1.7	4.8	1.8	3.9	1.9	3.5	1.1	3.2	1.6	2.1	1.8	4.9
5	0.5	2.2	0.7	3.2	0.1	0.7	0.6	2.4	0.6	2.1	0.2	2.8	0.2	1.0	0.3	0.5	0.4	2.0
6	0.1	0.4	0.1	0.3	0.1	0.8	0.0	1.3	0.3	0.1	0.0	0.0	0.1	0.4	0.0	0.0	0.1	0.4
7	0.0	0.3	0.0	0.4	0.0	0.1	0.1	0.1	0.0	0.4	0.0	0.0	0.0	0.0	0.0	0.0	0.0	0.2
8	0.0	0.0	0.0	0.0	0.0	0.0	0.0	0.0	0.0	0.0	0.0	0.0	0.0	0.0	0.0	0.0	0.0	0.0

^a^Maximum number of problems was 8 (1 daily smoking man in 2007)

## Discussion

### Main findings

Consistent with the findings of other national surveys, our results show a considerable decline in the prevalence of daily smoking in the adult Norwegian population over the past 14 years. We also found a falling trend in individual and combined material and lifestyle problems during the same period, especially among occasional and non-smokers combined. Simultaneously, a higher accumulation of problems among daily smokers than among occasional and non-smokers combined occurred. However, the differences in problems per smoking status group did not increase as hypothesised, except for the selected dietary behaviours.

### Comparison with the general population in Norway

From 1999 to the present, the average standard of living in Norway has improved, with both real income and purchasing power increasing considerably. Simultaneously, the occurrence of physical inactivity and unhealthy diet was lower in 2013 than in 1999 [45]. On the other hand, meeting friends and subjective perceptions of good health have not changed much, or might have even worsened to some extent, since the late 1990s in Norway [45, 46]. Our findings are in line with this, as we found little or no change in the categories “small social network” and “poor self-rated health” in the period investigated.

According to Statistics Norway, daily smoking has been declining for decades, and the findings of the current study only serve to corroborate this view. According to national statistics, 15 % of the male population and 14 % of the female population in Norway smoked every day in 2013 [47]. These figures are only marginally higher than the results for 2013 in the current study, which were 11 and 13 % for men and women, respectively.

The only potentially problematic behaviour category that has increased in our study from 1999 to 2013 is frequent drinking, as defined by the respondents. This is partially in agreement with the trends in the general population in Norway [48]. Total alcoholic beverage sales per annum in Norway rose between the early 1990s and around 2008–09, when a decline set in until 2013. However, the sale of alcohol in the form of wine rose steadily during the entire period. In addition, both tax-free sale and cross-border trade in wine and beer increased in recent years, as did the number of government-owned *Vinmonopol* retail stores and licensed premises, such as bars and restaurants [48]. Thus, alcohol seems to be more available to the general public than it was a few years ago. In contrast to the other categories of poor health behaviour included in the current study, drinking is socially accepted, and its social status is not as low as poor dietary habits and physical inactivity [27]. Even if the rate of frequent drinking among daily smokers is well in excess of that of occasional and non-smokers combined for each of the years studied, our results indicate a similar increase in the prevalence of frequent drinking among daily smokers and occasional and non-smokers combined in the past few years. Note that frequent drinking (as defined by the respondents) does not necessarily represent an unhealthy drinking habit. However, in a Norwegian context, where binge drinking is the norm, high average drinking frequencies are more likely to be a proxy for a problematic drinking pattern than in countries with “continental” drinking habits [49].

The only problem category where the differences between the groups of daily smokers and others actually widened during these years was poor dietary habits. The indicators of a poor diet that we studied showed an improvement in the group of occasional and non-smokers combined, but remained constant among daily smokers. In general, the diets of smokers tend to be poorer than those of non-smokers [50]. The average diet in Norway has improved during the 2000s [51], with a higher intake of fruit and vegetables and lower consumption of sugar and sugar-sweetened soft drinks. However, intake of meat and processed meat (as in fast foods) has been on the rise since the early 2000s. One reason why fast food intake seems to have fallen in the present sample might be that fast food items bought at snack bars is not as socially acceptable; young people in particular seem to prefer healthy fast food nowadays [52].

Smokers tend to attach less importance to health values than non-smokers, which may explain not only why they continue to smoke, but also why they are less concerned with healthy diets [51, 53, 54]. Appreciation of health values is also associated with class: while the middle classes often consider themselves and others in terms of their health-orientated behaviour, this is not necessarily the case among the working class, who often look at health and the body as private matters and as being more susceptible to luck/bad luck [55]. For many working class smokers with elementary education, smoking may therefore have a different meaning than for middle class groups.

Among both daily smokers and the group of occasional and non-smokers combined in our study, the numbers admitting to never exercising declined significantly, and approximately by the same degree, from 1999 to 2013. This is in line with findings in the general population in Norway between 2001 and 2007 [47, 48]. Vigorous physical activity and heavy smoking are negatively associated among adult men and women in Norway [50], while research investigating the associations between inactivity and smoking is missing. Unlike poor dietary factors, the difference between the smoking groups’ respective scores on physical inactivity did not change with time.

Lifestyle factors might arguably change faster and differently than basic material factors, which are more associated with demographic and socio-economic position. While work, housing and income are heavily dependent on structural and political conditions and welfare arrangements, lifestyle factors are, if not entirely unconstrained, at least more open to individual choice and values.

### To what extent are Norwegian daily smokers marginalised?

Our findings do not agree with the results of a comparable English study, nor do they evidently support the “marginalisation of smokers” thesis. In the English study, disadvantage (using some of the same indicators as we used, such as rented accommodation and unemployment) declined among non-smokers, but not among smokers [17]. In our study, disadvantage decreased in both groups or did not change in any of the smoking groups. Admittedly, transferring the findings of an English study to Norwegian conditions could be questionable, not least because of differences in the welfare arrangements of the two countries. The distribution of problems and resources may therefore vary, as might the absolute levels of problems and resources, too. There are also differences in social inequalities, with Norway being one the most homogeneous countries in the world. Compared to the rest of Europe, Norway is homogeneous not only in terms of material situation [56], but also in well-being and cultural identity [57]. Characterised by the “Nordic welfare model”, the level of affluence is high and economic differences between groups are small [58].

Even if daily smokers are in a generally less privileged situation than occasional smokers and non-smokers, the differences are not large and they do not increase over time. This indicates that the relative situation of daily smokers is not worsening; as a group they have no more problems in 2013 (when they constitute about 15 % of the population) than they had in 1999 (when daily smoking prevalence was about 30 %). While this consistency may have something to do with what in an international context may be characterised as Norwegian affluence, it also indicates that the growing “marginalisation of smokers” among the public (which is what we have studied here, and which we only find minor support for) is a different sort of question than the overrepresentation of smokers in marginal problem groups (which we have not studied here). The hardening hypothesis has also been questioned, and a recent study of 32 countries (US and EU) suggests that the remaining smoker population is in fact softening, not hardening [59].

### Limitations

#### Response rate

The low response rate of the current study raises concerns about the representativeness of the sample, and the validity of the results. The wide range of societal issues covered in the survey, of which some might appear complicated to citizens who do not follow politics closely, as well as the sheer magnitude of the questionnaire, might indicate a lower response rate among lesser privileged groupings in society. If the relative size of lesser privileged groups increases more among smokers than non-smokers over time, and these subjects do not respond to surveys to a greater extent, the non-response in different smoking groups might change differently over time and introduce a greater non-response bias in 2013 than in previous years, such a bias must be considered when interpreting the findings. However, the trends found in daily smoking in this study resemble those found in other studies with higher response rates, so the analytical sample in the current study would appear to be reasonably unbiased. Also, comparisons of the sample applied here with other data sets with regard to other indicators than smoking status (such as housing and BMI), suggest that the sample is largely representative when it comes to public health indicators [30, 47, 48]. Even if the sample, like any household survey, is likely to underestimate the size of the most marginalised smokers (homeless people, drug addicts, people in prisons), it is less likely that this underestimation threatens the validity of the study.

#### Weighted data

Weighting data to increase the representativeness of the study sample may cause some problems. In the current study, weighting was based on gender, age and geographic region of the general Norwegian population 15 years of age and older. The independent and dependent variables used in our analyses were not used in the weighting, as correct levels of material problems and lifestyle factors in the population are unknown. Based on a few selected variables, our weighting might therefore result in an even more biased sample with regard to, for example, smoking, health behaviour and self-rated health than the un-weighted data sample. The weighted smoking rates in the current sample are somewhat lower than in the un-weighted, and also lower than the smoking rates in nationally representative samples in Norway. However, the falling trend in smoking found in the weighted data is similar to the findings of other studies in Norway. The reported differences between daily smokers and occasional and non-smokers combined with regard to health behaviours are also in line with findings from other studies [50, 60–62].

We also performed all our analyses on un-weighted data, to compare with the results presented here. In general, the latter results did not differ significantly from those using weighted data. As mentioned, the estimated prevalence of daily smoking was somewhat higher when using un-weighted data (around two percentage points for all years combined), otherwise the results were similar using the two different methods. The similarities of the results from weighted and un-weighted data in the current study indicate that our findings are valid.

#### Self-reporting

All factors used in the current analyses were obtained by self-reporting, which is vulnerable to recall bias and social desirability [63–65]. Desirable positions and health-promoting behaviour may be overestimated while unwanted positions/situations and unhealthy behaviours may be underestimated. The potential for over and underestimation may differ in the different smoking groups, and one must bear in mind the possibility of incorrect estimates of associations.

## Conclusion

In line with general developments in Norwegian society, the current study confirmed the decline in material and lifestyle problems from 1999 to 2013. The gap between daily smokers and the group of occasional and non-smokers combined, however, did not grow wider over the years, with the exception of poor dietary habits. Consequently, it seems that daily smokers as well as occasional and non-smokers have benefited from the progress that has been achieved in Norway in recent decades, and we found little evidence of a greater accumulation of material and lifestyle problems among daily smokers than among occasional and non-smokers, as hypothesised.
